# Selectivity Enhancement in Molecularly Imprinted Polymers for Binding of Bisphenol A

**DOI:** 10.3390/s16101697

**Published:** 2016-10-14

**Authors:** Noof A. Alenazi, Jeffrey M. Manthorpe, Edward P. C. Lai

**Affiliations:** Department of Chemistry, Carleton University, 1125 Colonel By Drive, Ottawa, ON K1S 5B6, Canada; noof0988@hotmail.com (N.A.A.); jeff.manthorpe@carleton.ca (J.M.M.)

**Keywords:** bisphenol A, diazomethane, non-specific binding sites, site-selective chemical modification, treated molecularly imprinted polymers

## Abstract

Bisphenol A (BPA) is an estrogen-mimicking chemical that can be selectively detected in water using a chemical sensor based on molecularly imprinted polymers (MIPs). However, the utility of BPA-MIPs in sensor applications is limited by the presence of non-specific binding sites. This study explored a dual approach to eliminating these sites: optimizing the molar ratio of the template (bisphenol A) to functional monomer (methacrylic acid) to cross-linker (ethylene glycol dimethacrylate), and esterifying the carboxylic acid residues outside of specific binding sites by treatment with diazomethane. The binding selectivity of treated MIPs and non-treated MIPs for BPA and several potential interferents was compared by capillary electrophoresis with ultraviolet detection. Baclofen, diclofenac and metformin were demonstrated to be good model interferents to test all MIPs for selective binding of BPA. Treated MIPs demonstrated a significant decrease in binding of the interferents while offering high selectivity toward BPA. These results demonstrate that conventional optimization of the molar ratio, together with advanced esterification of non-specific binding sites, effectively minimizes the residual binding of interferents with MIPs to facilitate BPA sensing.

## 1. Introduction

Bisphenol A (BPA) is a household name of which many consumers are aware [1]. It is classified by the U.S. Environmental Protection Agency as an endocrine disrupting compound and thus interferes with the action of natural hormones in the body that are responsible for human behaviour, development, homeostasis and reproduction [2,3,4]. It may be reasonably anticipated to be a human carcinogen in the breast and prostate due to its tumor promoting properties [5]. BPA is a synthetic monomer, extensively used in the production of epoxy resins, polysulfone thermoplastics, polycarbonate plastics and thermal paper receipts [6,7]. Worldwide, over five million metric tons of BPA are produced annually for use in a variety of products, including baby bottles, food containers and beverage cans [8,9]. BPA has been detected in rivers among top manufacturing competitiveness index countries [10,11,12,13,14,15]. There is a need to detect this toxic chemical in a variety of scenarios, including aquatic environments, sewage effluents, and non-dietary sources, as well as human urine and blood [16,17,18,19]. A robust experimental study has shown that developmental exposure of pregnant rats to 25 μg of BPA per kg body weight per day can cause adverse effects on fertility (decreased sperm count), neurodevelopment (masculinization of spatial learning in females) and lead to increased female body weight late in the life of their offspring [20]. This low-dose perinatal exposure can also affect mammary gland development in male and female offspring [21]. A liquid chromatography-tandem mass spectrometry analytical method was used to directly and simultaneously measure unconjugated BPA, BPA glucuronide and BPA sulfate in the urine of pregnant women in their second trimester [22]. Near universal and high exposure to BPA and its conjugated metabolites was found among pregnant women, raising additional concerns for their fetal development. Creatinine-standardized BPA concentrations decreased over childhood, from 1 to 8 years of age [23]. BPA concentrations were positively associated with consuming food stored or heated in plastic, consuming canned food and beverages, and handling cash register receipts.

One efficient technique to detect BPA is to use a molecularly imprinted polymer (MIP) as a recognition element in separation systems, chemical assays, colorimetric devices, electrochemical sensors, or fluorescent sensors [24,25,26,27,28,29,30,31]. MIP synthesis creates imprinted sites that are similar in size and shape to BPA. These imprinted sites are capable of behaving very selectively and efficiently in rebinding the target molecules [32,33]. MIPs are inexpensive to produce for industrial applications [34]. A potentially very important problem with MIPs is the presence of non-specific binding sites, as this often limits the use of MIPs in sensor applications [35,36]. Recent research has reported relative binding efficiencies of 0.46–0.48 for BPA structural analogs such as 2,4-dichlorophenol and p-nitrophenol [37]. Selectivity coefficients of 2.5–2.7 were observed for hydroquinone, 8-hydroxy quinolone, phenol and 4-nitrophenol, all of which have similar structures to the BPA molecule [38]. Other potential competitor interferents that are commonly found in water include pharmaceuticals and personal care products. Hence, there is a clear and pressing need to develop techniques that either minimize the formation of non-specific sites during the synthesis of MIPs or allow for blocking of non-specific binding sites after the polymer preparation. For the most demanding sensor applications, both techniques can be combined to enhance the selectivity of binding only the target analyte molecule.

This study examined both the site-selective chemical modification (SSCM) technique, which employs partial esterification of the residual carboxylic acid groups using diazomethane [39,40,41], and optimization of the template to functional monomer to cross-linker molar ratio [42,43]. The aim was to eliminate non-specific binding sites in the MIP. The binding selectivity of diazomethane-treated molecularly imprinted polymers (TMIPs) and non-treated MIPs, as well as diazomethane-treated non-imprinted polymers (TNIPs) and non-treated NIPs, toward BPA was evaluated, by capillary electrophoresis (CE) with ultraviolet (UV) detection, against three widely prescribed pharmaceutical compounds (baclofen, diclofenac and metformin) which are abundantly distributed in environment waters [44]. In our previous article [40] we tested cross reactivity with the same compounds and also used competitive CE-UV binding tests. The present article contains more data on the systematic optimization of the TMIP to demonstrate an improvement of selective binding results.

## 2. Materials and Methods

### 2.1. Materials

BPA, baclofen (BFN), diclofenac sodium salt (DFC), diethyl ether, ethylene glycol dimethacrylate (EGDMA), methacrylic acid (MAA) and metformin hydrochloride (MF) were purchased from Sigma-Aldrich (Oakville, ON, Canada). 2,2′-Azobis(2-methylpropionitrile) (AIBN) was obtained from Pfaltz and Bauer (Waterbury, CT, USA). Triethylamine (TEA) was acquired from Fluka (Buchs, Switzerland). Sodium phosphate dibasic was supplied by Fisher Scientific (Fair Lawn, NJ, USA). All chemicals were used as received without any further purification except diethyl ether, which was distilled from lithium aluminum hydride. High performance liquid chromatography (HPLC)-grade methanol and acetonitrile, and spectro-grade acetone, were all purchased from Caledon (Georgetown, ON, Canada).

### 2.2. Apparatus and Analytical Instruments

A diazomethane generator kit (“Mini Diazald apparatus” (Aldrich part # Z108898), Clear-Seal flask (Aldrich part # Z100358)); and a separatory funnel with polytetrafluoroethylene stopcock and Clear-Seal joint (Aldrich part # Z100382) was obtained from Sigma-Aldrich. CE-UV analysis was performed on a laboratory-built system [40]. The background electrolyte (BGE) was composed of 10 mM Na_2_HPO_4_ in deionized distilled water (DDW) to attain pH 7.5 ± 0.2. A Shimadzu liquid chromatography system with UV detection was used to determine the hydrophobicity of BFN, BPA, DFC and MF using acetonitrile/methanol/DDW (1:1:1) as the mobile phase at a flow rate of 0.8 mL/min through a Phenomenex reversed phase C18 column (4.6 mm × 250 mm, 4 μm packing material).

### 2.3. Preparations of MIP and NIP Particles 

MIP_1_ through MIP_9_ were prepared using different molar ratios of BPA to MAA while keeping EGDMA constant, as listed in supplementary Table S1. All MIPs were prepared by following a previously reported procedure [40]. For direct comparison of binding test results, non-imprinted polymers (NIPs) were prepared analogously but in the absence of BPA.

### 2.4. Diazomethane Preparation and Treatment of MIP and NIP Particles

**Scheme 1:**
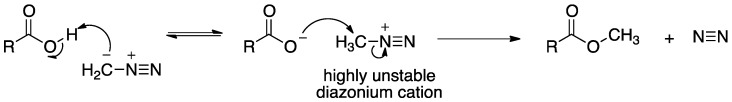
Methylation of carboxylic acid functional group with diazomethane. R represents the molecularly imprinted polymer or non-imprinted polymer matrix.

Due to the potentially explosive and toxic nature of diazomethane, this reaction must be performed behind a safety shield in a fume hood. Diazomethane was prepared from *N*-methyl-*N*-nitroso-*p*-toluenesulfonamide (Diazald^®^), Reference [45] following a literature procedure [46], using an Aldrich mini Diazald apparatus, as detailed elsewhere [40]. The final solution of CH_2_N_2_ gas, dissolved in diethyl ether, was protected from shock and stored in a freezer (at –15 °C). The NIP or unwashed MIP particles (670 mg) were dried overnight in an oven at 50 °C and suspended in diethyl ether (50 mL). An ethereal diazomethane solution was added to the suspension of particles in order to methylate their carboxylic acid groups (Scheme 1). BPA was then removed from the TMIPs with 5% TEA in methanol.

### 2.5. Competitive CE-UV Binding Tests

MIP, TMIP, NIP or TNIP particles were suspended in 10 mM Na_2_HPO_4_ (pH 7.5) BGE containing 100–200 ppm of BPA, BFN, DFC and MF to evaluate their bindings under sonication for 20 min to assist with uniform dispersion of particles throughout the suspension and rapid access of molecules to all binding sites. After the particles were precipitated by centrifugation at 10,000 rpm for 10 min, the concentrations of BPA, BFN, DFC and MF in the supernatant were determined by CE-UV analysis. All binding tests were carried out in duplicate, and percentage binding was calculated as 100% × (peak area before binding–peak area after binding)/peak area before binding.

### 2.6. Electrophoretic Mobility of Polymers

The ionic charge states of MIPs, NIPs, TMIPs and TNIPs particles were determined by CE-UV analysis after mesityl oxide was also analyzed as the neutral marker to provide a reference migration time (*t_ref_*). The electrophoretic mobility *μ_ep_* of each polymer was calculated as

*μ_ep_* = (*L_tot_ L_d_*/*V*) (1/*t_p_* − 1/*t_ref_*)

where *L_d_* is the capillary length from the inlet to the detector, *V* is the high voltage applied across the total capillary length *L_tot_*, and *t_p_* are the migration times of the particles, respectively.

## 3. Results and Discussion

### 3.1. Optimization of Template to Functional Monomer Molar Ratio

**Figure 1:**
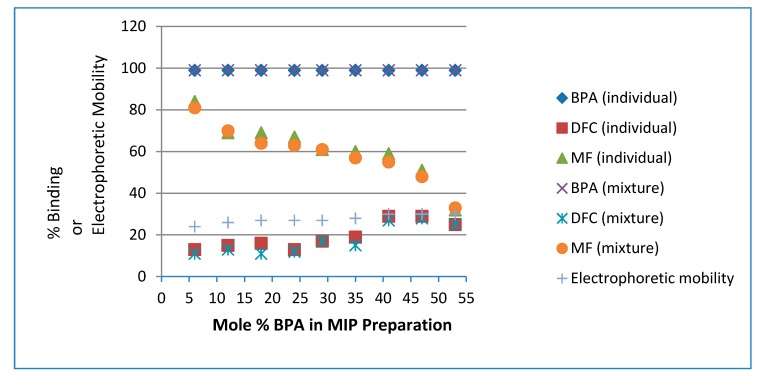
Percent binding results of bisphenol A (BPA), diclofenac (DFC) and metformin (MF) (individually and in mixture) and electrophoretic mobility for each MIP prepared with a different mole % of BPA. For clarity, error bars are represented by the size of symbols.

Although other functional monomers (e.g., 4-vinylpyridine and *N*-methacryloyl-l-phenylalanine) have been used to prepare MIPs for specific recognition of BPA in sensor applications, they either exhibited a maximum response at a high pH of 10 or required special synthesis in an organic chemistry research lab. Hence a commercially available monomer, MAA, with a pKa value of 4.66 was deemed preferable in the present work. Previous work by another research group had the molar ratio of BPA:MAA varied from 1:1 up to 1:8; the best MIP performance (i.e., the largest BPA peak area) was attained at a molar ratio of 1:4 but a large amount of 4-phenylphenol was also bound [47]. The molar ratio of BPA to MAA in our present work was varied from 1:9 down to 9:1 in an attempt mainly to reduce non-specific binding sites in the MIP. This straightforward approach could be an effective method to enhance the selectivity of MIP toward BPA without the use of diazomethane treatment. All MIP and NIP particles were tested for their BPA selectivity, using DFC and MF as negatively and positively charged interferents, respectively. As the molar ratio of BPA to MAA increases, the binding selectivity of MIP for BPA, defined as the % binding for BPA relative to those for DFC and MF, is enhanced without compromising the 99% binding of BPA (as can be seen in Figure 1). DFC exhibits a slightly higher affinity towards MIP_7_, MIP_8_ and MIP_9_ than the other MIPs, probably due to stronger hydrophobic interaction with these former MIP particles as the ratio of MAA to EGDMA and BPA decreases and the polymer becomes less negatively charged. Obviously, MIP_9_ shows the lowest binding affinity toward MF, both individually and in mixture, which, in conjunction with the electrophoretic mobility data, indicates that this polymer has the lowest negative charge among the MIPs. All percent binding results of BPA, DFC and MF (individually and in mixture), as well as electrophoretic mobility, are presented in Figure 1 for each MIP prepared with a different BPA mole %. A significant decrease in % binding for MF and slight increase in % binding for DFC are clearly seen when going from MIP_1_ to MIP_9_, all of which exhibit a constant 99% binding for both BPA (individual) and BPA (mixture).

For comparison purposes, the study tested the binding affinity of BPA (individually and in the three-component mixture) toward NIPs. When the ratio of MAA to EGDMA decreased upon going from NIP_1_ to NIP_9_, as shown in supplementary Table S2, the binding affinity of DFC and BPA toward the NIPs increased due to elevated hydrophobic interactions and in the case of DFC, decreased electrostatic repulsion—that is the same trend as with MIPs. The binding affinity of MF decreased from 70% ± 1% to 50% ± 1% individually and from 73% ± 1% to 56% ± 1% in mixture. While the direction of this trend is consistent with the results in MIPs, the magnitude is far less pronounced. These decreases observed with the NIPs were neither so significant in range (from highest to lowest) nor related to the electrophoretic mobility values (which indicate a fairly constant electronic charge to particle size ratio for all of the polymers). The apparently higher values of electrophoretic mobility for NIPs do not necessarily mean higher electronic charges, but rather smaller sizes than MIPs, due to the absence of imprinted cavities in the NIPs (vide infra). 

### 3.2. Esterification of MIPs with Diazomethane 

**Figure 2:**
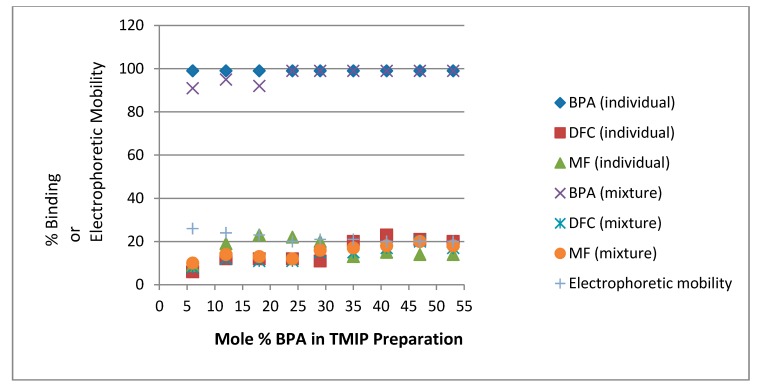
Percent binding results of BPA, DFC and MF (individually and in mixture) and electrophoretic mobility for each diazomethane-treated molecularly imprinted polymers (TMIP) prepared with a different mole % of BPA. For clarity, error bars are represented by the size of symbols.

To enhance selective binding with the target analyte molecules, diazomethane treatment was chosen as an alternative or combined approach to reduce or eliminate non-specific binding sites in the MIPs. In Figure 2, all TMIPs showed diminished binding affinity toward MF, a positively charged compound. In particular, TMIP_1_ revealed the lowest binding affinity toward MF, at 9% ± 1% individually and 10% ± 1% in mixture.

Unlike our MIPs, BPA binding affinity toward TMIP_1_ in the three-component mixture shows a slightly lower binding affinity of 91% ± 1%. As the molar ratio of BPA is increased in Figure 2, the binding affinity of TMIPs is enhanced up to 99% (both individually and in the mixture for TMIP_4_ to TMIP_9_), presumably as a result of an increase in the number of specific binding sites. Efforts to determine the BPA binding affinity toward TMIP_10_ (10:0:7 BPA:MAA:EGDMA) were to no avail because the particles could not be uniformly dispersed in water samples. All TMIPs show less binding affinity than MIPs toward DFC, particularly TMIP_1_ at only 6% ± 1% individually and 8% ± 1% in mixture. TMIPs also show significantly less binding affinity toward MF when compared to MIPs, which indicates a decrease of electrostatic interactions between MF and the polymer. Comparing the binding affinity of MF in the three-compound mixture, TMIP_1_ demonstrates the most dramatic decrease, dropping to 10% from 73% for MIP_1_. All binding results are presented in Figure 2 for each TMIP. The separation of BPA data points at the top from those for DFC and MF near the bottom is a very distinctive and impressive illustration of the high selectivity offered by TMIPs toward the target analyte. One rationale for using such a large excess of template (9:1 template: monomer to synthesize TMIP_9_) is that EGDMA can act as both a cross-linker and a functional monomer to form BPA:EGDMA complexes in the pre-polymerization mixture. Comparing with our previous results obtained using a molar ratio of 1:8:7 for BPA:monomer:crosslinker in the preparation of TMIP [40], the present selectivities are better. Noticeably, all TMIPs prepared using 6 to 24 mole % of BPA show less binding affinity toward DFC than the previous result of 16%; particularly the TMIP prepared using 6 mole % of BPA bind DFC at only 6% individually and 8% in mixture.

**Figure 3:**
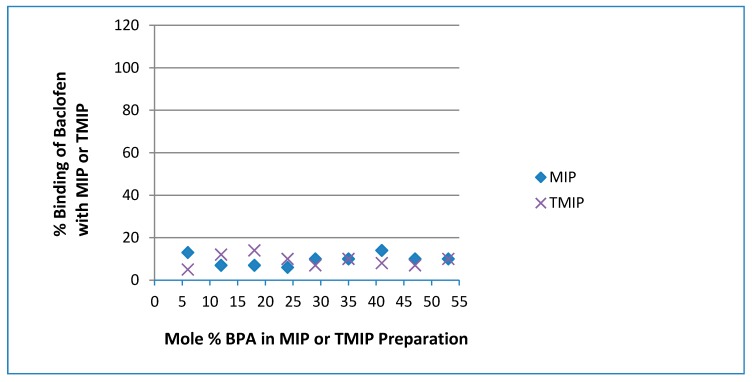
Percent binding results of BFN (individually) for each MIP and TMIP prepared with a different mole % BPA. For clarity, error bars are represented by the size of symbols.

In comparison with TMIPs, TNIPs show higher non-specific binding affinities toward MF and DFC (supplementary Table S3). Interestingly, many TNIPs also have a significantly higher binding affinity toward BPA than NIPs. One plausible explanation is that the ester functional group affords stronger interaction with BPA than the carboxylate functional group. This is an important finding in the sense that, by converting acids into esters, the number of binding sites for BPA was increased in all TNIPs. The increased proportion of EGDMA (which is also an ester) could potentially be beneficial in shaping the binding sites. This would help explain why TMIP_9_ performed better than TMIP_1_ in binding with BPA despite a decrease of the MAA monomer molar ratio from 9 (for TMIP_1_) to 1 (for TMIP_9_). Also, when looking at the supplementary Table S2 and Table S3, every NIP or TNIP shows a much better affinity for BPA than the cross reactive compounds. So, the imprinting factors are apparently not very high. However, as pointed out by Vasapollo et al in their review article, an even better MIP evaluation than imprinting factor is the selectivity for different molecules [48]. The imprinting factor must be considered along with selectivity results since a good binding result may just be due to particular physicochemical properties of the target molecule rather than the presence of specific imprinted sites. The non-specific binding properties of MIP, NIP, TMIP and TNIP particles were further investigated by CE-UV analysis of baclofen (BFN), which is a derivative of γ-aminobutyric acid (primarily used to treat spastic movement disorders). Although its strongest acidic pK_a_ is 3.9 and its strongest basic pK_a_ is 9.8 [49], the BFN peak was not separable from the BPA peak and hence appeared to be a neutral compound. As summarized in supplementary Table S4, the binding results for BFN are unexpected—significantly lower than those discussed above for BPA and fairly similar to those for diclofenac sodium. It is well known that BPA has a pK_a_ value between 9.9 and 11.3 [50] and an octanol–water partitioning coefficient (K_ow_) value of 103.4 [51]. The latter property indicates that BPA is a significantly more hydrophobic compound than BFN, which has a log partition coefficient (log P) value of −0.80 ± 0.02 [48] or a partition coefficient (P) value of 0.16. Figure 3 presents the percent binding results of BFN for each MIP and TMIP prepared with a different mole % of BPA. On a full binding scale from 0% to 100%, the interferences of baclofen binding with the MIPs and TMIPs were low and the differences among them were insignificant. Despite the scatter of data points between 5% binding and 20% binding, BFN behaves similarly to DFC (see Figure 1 and Figure 2), which has a different pK_a_ value of 4.0 [52] and a larger molecular size. Their non-specific binding mechanisms (including hydrophobic and ionic interactions) are worthy of more systematic investigation.

**Figure 4:**
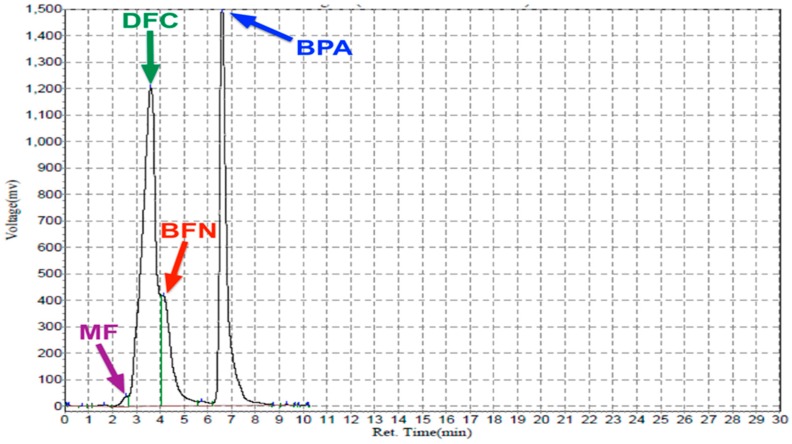
HPLC analysis of a mixture of MF (200 ppm) at 2.5 min, DFC (200 ppm) at 3.6 min, BFN (200 ppm) at 4.1 min and BPA (100 ppm) at 6.6 min. Mobile phase: CH_3_CN/MeOH/DDW (1:1:1 v/v); flow rate, 0.8 mL/min; UV detection at 200 nm.

In order to gain a better understanding of the non-specific binding mechanisms, high-performance liquid chromatography with UV detection was used to analyze standard solutions of BFN, BPA, DFC and MF (individually or in mixture) on a reverse-phase column. The retention time of each organic compound, analyzed individually as summarised in supplementary Table S5, would serve as a direct indication of its hydrophobicity. As illustrated in Figure 4, their order of elution was MF first, DFC second, BFN third, and BPA last. This indicated that MF is the least hydrophobic and BPA is the most hydrophobic of the four compounds. The fact that the least hydrophobic compound was able to bind non-specifically with the MIPs (from 32% to 84% in Figure 1) emphasizes a real need to perform SSCM on the MIPs by treating them with diazomethane. Apparently, moderately hydrophobic compounds such as BFN and DFC bind non-specifically with the MIPs not more than 29% (see Figure 1), whether they are zwitterionic or negatively charged in aqueous samples. Site-selective chemical modification with diazomethane, however, can decrease their non-specific bindings down to 23% (see Figure 2). 

### 3.3. Average Size of MIPs and NIPs Vs TMIPs and NTIPs 

**Figure 5:**
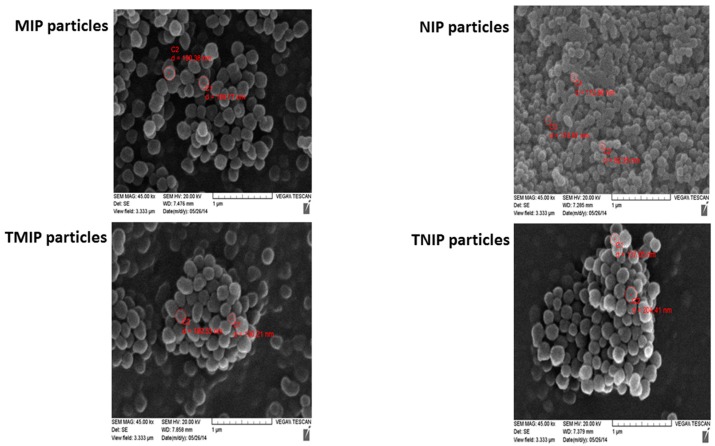
Scanning electron microscopy images of MIP, NIP, TMIP and TNIP particles.

**Figure 6:**
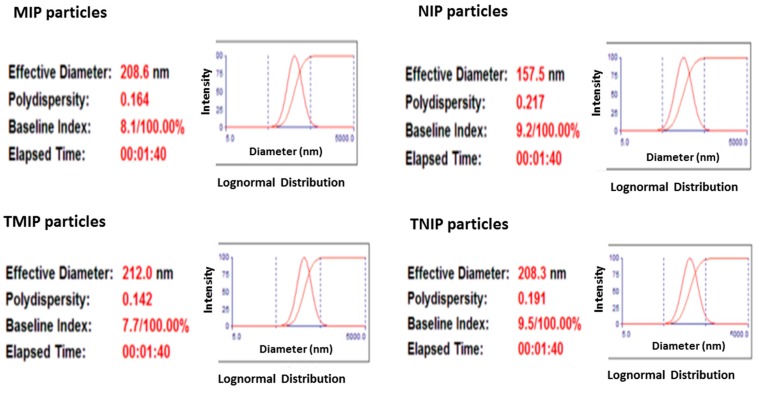
Dynamic light scattering analysis of MIP, NIP, TMIP and TNIP particles.

The average sizes of MIP, NIP, TMIP and TNIP particles were determined by scanning electron microscopy (SEM). The SEM images in Figure 5 show both the size and the size distribution of polymer particles. MIP particles were approximately 169–190 nm in diameter and NIP particles were approximately 112–115 nm. Similarly, TMIP particles were >180 nm in diameter and TNIP particles were >137 nm. These particle sizes were confirmed by dynamic light scattering (DLS) analysis shown in Figure 6 that reported a diameter of 208 ± 2 nm for MIP, 157 ± 1 nm for NIP, 212 ± 2 nm for TMIP, and 208 ± 1 nm for TNIP. Such similar sizes of the TMIP and TNIP particles are consistent with their electrophoretic mobility values presented in Figure 2 and supplementary Table S3.

## 4. Conclusions

Blocking non-specific binding sites of MIP with diazomethane treatment is a more efficient approach than optimization of the template to monomer to cross-linker molar ratio, to produce TMIPs that can significantly reduce potential interferences during the selective extraction of BPA in water analysis. The binding affinity of TMIP particles is up to 99%, higher than the 86.6%–95.5% recovery reported recently [37]. These materials can be readily applied as a recognition element in colorimetric, electrochemical or fluorescent sensors to detect BPA in environmental waters. Baclofen, diclofenac and metformin are good for use as model interferences to test all TMIP-based sensors for accurate BPA determination. For trace environmental analysis, a relatively large volume of water can be sampled in order to minimize the problems associated with template bleeding. Conventional sorbent materials cannot easily handle any large volume of environmental water (to concentrate a target compound at trace levels) due to rapid saturation of their non-specific binding sites by the diverse population of competitor interferents present. Although diazomethane treatment of NIP particles can produce TNIP particles with an enhanced binding affinity for BPA, their non-specific binding with MF and DFC is undesirably double that of TMIP particles; this makes TMIP particles definitively superior and advantageous over TNIP particles in chemical sensor applications where BPA must be detected in water containing interferents at significant concentrations. Our TMIP nanoparticles demonstrated a specific affinity for BPA, comparable to the colloidal nanoparticles functionalized by a BPA aptamer (with a dissociation constant K_d_ of 54 nM) [53]. Non-specific binding of competitor interferents due to hydrophobic interactions can be ameliorated by modifying aqueous samples with a small portion of organic solvent such as methanol. Further research is underway to study the TMIP material properties, binding isotherms, sorption capacity, porosity and robustness in our chemical sensor research. The study will include data on quantifying BPA in water and give the limit of quantification, thereby allowing readers to compare the merits of this strategy to regulatory guidelines for BPA analysis, as well as its utility in application to the analysis of other organic compounds of concern.

## Supplementary Materials

The following are available online at http://www.mdpi.com/1424-8220/16/10/1697/s1, Table S1: Preparation of MIPs using different molar ratios of BPA to MAA while keeping EGDMA constant, Table S2: Percent binding results for BPA, DFC and MF (individually or in mixture) and electrophoretic mobility for NIPs, Table S3: Percent binding results for BPA, DFC and MF (individually or in mixture) and electrophoretic mobility for various TNIPs, Table S4: Percent binding results for BFN (individually) with various MIPs, NIPs, TMIPs and TNIPs, Table S5: HPLC-UV peak heights, peak areas and retention times for BFN, BPA, DFC and MF.

## Abbreviations

The following abbreviations are used in this manuscript:

AIBN	2,2′-azobis(2-methylpropionitrile)
BFN	baclofen
BGE	background electrolyte
BPA	bisphenol A
CE	capillary electrophoresis
DDW	distilled deionized water
DFC	diclofenac sodium salt
DLS	dynamic light scattering
EGDMA	ethylene glycol dimethacrylate
HPLC	high performance liquid chromatography
K_d_	dissociation constant
K_ow_	octanol-water partitioning coefficient
MAA	methacrylic acid
MF	metformin hydrochloride
MIP	molecularly imprinted polymer
NIP	non-imprinted polymer
P	partition coefficient
SSCM	site-selective chemical modification
SEM	scanning electron microscopy
TEA	triethylamine
TMIP	treated molecularly imprinted polymer
TNIP	treated non-imprinted polymer
UV	ultraviolet
