# Supplementary material for: Selectivity Enhancement in Molecularly Imprinted Polymers for Binding of Bisphenol A
Source: Sensors (Basel). 2016 Oct 14;16(10):1697. doi: 10.3390/s16101697 (PMC5087485; doi:10.3390/s16101697)
Supplement: Supplementary file 1 [file sensors-16-01697-s001.pdf]

Supplementary Materials: Selectivity Enhancement in Molecularly Imprinted Polymers for Binding of Bisphenol A

Noof A. Alenazi, Jeffrey M. Manthorpe and Edward P. C. Lai

Table S1. Preparation of MIPs using different molar ratios of BPA to MAA while keeping EGDMA constant.

Molecularly Imprinted Polymer	Molar Ratio			Mole % BPA
	BPA (mmol)	MAA (mmol)	EGDMA (mmol)	
MIP_1	1	9	7	6
MIP_2	2	8	7	12
MIP_3	3	7	7	18
MIP_4	4	6	7	24
MIP_5	5	5	7	29
MIP_6	6	4	7	35
MIP_7	7	3	7	41
MIP_8	8	2	7	47
MIP_9	9	1	7	53

Table S2. Percent binding results for BPA, DFC and MF (individually or in mixture) and electrophoretic mobility for NIPs.

NIPs (MAA:EGDMA Molar Ratio)	% Binding (Individual)			% Binding (Mixture)			Electrophoretic Mobility (10^{-9} $m^2 \cdot V^{-1} \cdot s^{-1}$)
	BPA	DFC	MF	BPA	DFC	MF	
NIP_1 (9:7)	80 ± 1	25 ± 1	70 ± 1	75 ± 1	25 ± 1	73 ± 1	29
NIP_2 (8:7)	88 ± 1	26 ± 1	73 ± 1	75 ± 1	18 ± 2	67 ± 1	27
NIP_3 (7:7)	91 ± 1	29 ± 1	71 ± 1	92 ± 1	33 ± 2	74 ± 1	28
NIP_4 (6:7)	92 ± 1	31 ± 1	62 ± 1	92 ± 1	25 ± 2	60 ± 1	29
NIP_5 (5:7)	91 ± 1	29 ± 2	75 ± 1	96 ± 1	32 ± 1	62 ± 1	29
NIP_6 (4:7)	90 ± 1	25 ± 1	69 ± 1	97 ± 2	28 ± 3	58 ± 1	28
NIP_7 (3:7)	99 ± 1	32 ± 1	55 ± 2	99 ± 1	38 ± 2	58 ± 1	28
NIP_8 (2:7)	99 ± 1	37 ± 1	58 ± 1	99 ± 1	39 ± 1	54 ± 1	28
NIP_9 (1:7)	96 ± 1	40 ± 1	50 ± 1	99 ± 1	43 ± 1	56 ± 1	28

Table S3. Percent binding results for BPA, DFC and MF (individually or in mixture) and electrophoretic mobility for various TNIPs.

TNIPs	% Binding (Individual)			% Binding (Mixture)			Electrophoretic Mobility
(MAA:EGDMA Molar Ratio)	BPA	DFC	MF	BPA	DFC	MF	(10^{-9} $m^2 \cdot V^{-1} \cdot s^{-1}$)
$TNIP_1$ (9:7)	99 ± 1	18 ± 1	54 ± 1	99 ± 1	14 ± 1	41 ± 1	25
$TNIP_2$ (8:7)	99 ± 1	10 ± 1	27 ± 1	90 ± 1	14 ± 1	27 ± 1	24
$TNIP_3$ (7:7)	99 ± 1	16 ± 1	38 ± 1	90 ± 1	16 ± 1	30 ± 1	24
$TNIP_4$ (6:7)	99 ± 1	17 ± 1	30 ± 1	99 ± 1	19 ± 1	26 ± 1	23
$TNIP_5$ (5:7)	99 ± 1	19 ± 2	17 ± 1	99 ± 1	40 ± 1	36 ± 1	24
$TNIP_6$ (4:7)	99 ± 1	26 ± 1	30 ± 1	99 ± 1	26 ± 1	16 ± 1	24
$TNIP_7$ (3:7)	99 ± 1	24 ± 1	29 ± 1	99 ± 1	37 ± 1	22 ± 1	24
$TNIP_8$ (2:7)	99 ± 1	36 ± 1	27 ± 1	99 ± 1	30 ± 1	20 ± 1	23
$TNIP_9$ (1:7)	99 ± 1	45 ± 1	31 ± 1	99 ± 1	54 ± 1	30 ± 1	22

Table S4. Percent binding results for BFN (individually) with various MIPs, NIPs, TMIPs and TNIPs.

**Baclofen % Binding (Individual)**			
**MIPs**	**NIPs**	**TMIPs**	**TNIPs**
MIP_1= 13 ± 3	NIP_1 = 14 ± 1	$TMIP_1$ = 5 ± 2	$TNIP_1$ = 8 ± 3
MIP_2= 7 ± 3	NIP_2 =16 ± 2	$TMIP_2$ = 12 ± 2	$TNIP_2$ = 10 ± 3
MIP_3 = 7 ± 3	NIP_3 = 16 ± 2	$TMIP_3$ = 14 ± 2	$TNIP_3$ = 7 ± 2
MIP_4 = 6 ± 3	NIP_4 = 17 ± 2	$TMIP_4$ = 10 ± 1	$TNIP_4$ = 4 ± 3
MIP_5 =10 ± 2	NIP_5 = 16 ± 2	$TMIP_5$ = 7 ± 3	$TNIP_5$ = 4 ± 3
MIP_4 = 10 ± 2	NIP_6 = 10 ± 3	$TMIP_6$ = 10 ± 2	$TNIP_6$ = 6 ± 2
MIP_3 =14 ± 2	NIP_7 = 7 ± 2	$TMIP_7$ = 8 ± 2	$TNIP_7$ = 6 ± 3
MIP_2 = 10 ± 1	NIP_8 = 7 ± 3	$TMIP_8$ = 7 ± 2	$TNIP_8$ = 8 ± 3
MIP_2 = 10 ± 1	NIP_9 = 9 ± 2	$TMIP_9$ = 10 ± 2	$TNIP_9$ = 12 ± 3

Table S5. HPLC-UV peak heights, peak areas and retention times for BFN, BPA, DFC and MF.

**Concentration (ppm)**	**Peak Area**	**Peak Height**	**Retention Time (min)**
MF (200)	679,024	38,547	2.5
DFC (200)	49,417,648	1,205,569	3.6
BFN (200)	12,274,367	417,392	4.1
BPA (100)	32,571,576	1,487,642	6.6
